# Fabrication and characterization of mechanically competent 3D printed polycaprolactone-reduced graphene oxide scaffolds

**DOI:** 10.1038/s41598-020-78977-w

**Published:** 2020-12-17

**Authors:** Amir Seyedsalehi, Leila Daneshmandi, Mohammed Barajaa, John Riordan, Cato T. Laurencin

**Affiliations:** 1 Connecticut Convergence Institute for Translation in Regenerative Engineering, UConn Health, 293 Farmington Avenue, Farmington, CT 06030 USA; 2 Raymond and Beverly Sackler Center for Biomedical, Biological, Physical and Engineering Sciences, UConn Health, Farmington, CT 06030 USA; 3 Department of Biomedical Engineering, University of Connecticut, Storrs, CT 06269 USA; 4 Department of Orthopaedic Surgery, UConn Health, Farmington, CT 06030 USA; 5 Institute of Materials Science, University of Connecticut, Storrs, CT 06269 USA; 6 Department of Materials Science and Engineering, University of Connecticut, Storrs, CT 06269 USA; 7 Department of Chemical and Biomolecular Engineering, University of Connecticut, Storrs, CT 06269 USA

**Keywords:** Biomedical engineering, Biomedical materials

## Abstract

The ability to produce constructs with a high control over the bulk geometry and internal architecture has situated 3D printing as an attractive fabrication technique for scaffolds. Various designs and inks are actively investigated to prepare scaffolds for different tissues. In this work, we prepared 3D printed composite scaffolds comprising polycaprolactone (PCL) and various amounts of reduced graphene oxide (rGO) at 0.5, 1, and 3 wt.%. We employed a two-step fabrication process to ensure an even mixture and distribution of the rGO sheets within the PCL matrix. The inks were prepared by creating composite PCL-rGO films through solvent evaporation casting that were subsequently fed into the 3D printer for extrusion. The resultant scaffolds were seamlessly integrated, and 3D printed with high fidelity and consistency across all groups. This, together with the homogeneous dispersion of the rGO sheets within the polymer matrix, significantly improved the compressive strength and stiffness by 185% and 150%, respectively, at 0.5 wt.% rGO inclusion. The in vitro response of the scaffolds was assessed using human adipose-derived stem cells. All scaffolds were cytocompatible and supported cell growth and viability. These mechanically reinforced and biologically compatible 3D printed PCL-rGO scaffolds are a promising platform for regenerative engineering applications.

## Introduction

Scaffolds are three-dimensional (3D) constructs that serve as temporary templates and provide cells with an appropriate environment for tissue regeneration and formation. In engineering these temporary templates, there are several characteristics that must be considered and fulfilled. They should be able to withstand physical loads, be mechanically compatible with the surrounding environment, and serve as structural support systems for tissue regeneration to take place. They should be biocompatible and safe. Ideally, they should be able to support the adhesion, spreading, growth, proliferation, and differentiation of cells. Over time, they should degrade and release by-products that are safe and harmless to the body. Additionally, they should be highly porous and have an interconnected pore network that supports the growth of cells and transport of metabolic wastes^1–4^.

A myriad of techniques are available to fabricate scaffolds including solvent casting^5^, freeze casting^6^, freeze drying^7^, electrospinning^8^, gas foaming^9^, particulate-leaching^10^, melt molding^11^, phase separation^12^, and self-assembly^13^ and sol–gel methods^14^. However, most techniques are limited by their poor control over scaffold design, architecture, pore network and pore size. Additionally, these techniques are restricted in their ability to provide consistency and repeatability in producing scaffolds with the same design parameters^15,16^.

Additive manufacturing or 3D printing is a state-of-the-art technology that enables the fabrication of complex geometries with high accuracy and control. Constructs are created through a layer-by-layer deposition process, with subsequent layers being fused to produce the final structure. 3D printing overcomes the limitations of conventional scaffold fabrication techniques in that it can produce well-defined architectures that are controlled and consistent in their outer geometry, internal architecture, strand size, and pore size and distribution^17–19^. The 3D printing fabrication techniques are generally categorized into inkjet printing, laser-assisted printing and extrusion-based printing, with each technique offering its own unique advantages^17^. Extrusion-based 3D printing in particular is the most versatile technique that can be used to print a wide array of materials including polymers, ceramic pastes, hydrogels, and cell-laden bioinks. It offers high precision and fidelity^20^ and can be readily modified depending on the material and solidification technique^17,20^.

Biodegradable synthetic polymers such as poly(lactic-co-glycolic acid) (PLGA), polylactic acid (PLA), and polycaprolactone (PCL) are among the most widely used polymers for scaffold fabrication due to their biocompatibility, biodegradability, processability and tunable mechanical properties^21^. PCL in particular is an FDA-cleared aliphatic semi-crystalline polyester that is readily manufactured and manipulated for a wide range of applications^22^. Its prolonged degradation rate can be used to produce biodegradable devices for long-term use but can also be manipulated to tune the polymer’s biodegradation rates^23^. PCL has excellent solubility and processability in a large number of organic solvents, which make it suitable for use in different fabrication techniques and for diverse tissue types^21^. Additionally, it has a glass transition temperature of − 60 °C, and a melting temperature of 55–60 °C, and thus is one of the most preferred polymers for extrusion-based 3D printing^24^. Despite its promising features, however, the hydrophobicity and lack of functional groups in PCL pose challenges for its usage in biomedical applications.

Graphene is a single atomic layer of carbon atoms that are tightly packed into a 2D honeycomb structure. Its high specific surface area and extraordinary mechanical, electrical, thermal, and optical properties make it one of the most versatile materials for use in different industries and applications. The impressive properties of graphene and its derivatives known as the graphene family of materials (GFMs) have garnered much interest among scientists worldwide^25–27^. Graphene oxide (GO) is the oxidized form of graphene that is generated from the oxidation and exfoliation of graphite. The oxidation introduces oxygen functional groups that albeit improving the hydrophilicity and processability, act as defects within the planar structure of graphene and diminish its properties^28,29^. To remove some of the oxygen functional groups that disrupt the structure, reduced graphene oxide (rGO) is produced from the reduction of GO. rGO can be considered as an intermediate structure between the perfect graphene sheet and the oxidized GO structure that has a partial restoration of some of the properties that were lost due to oxidation^30^.

Composite structures of rGO with various polymers typically demonstrate improvements in their physicochemical and biological features^31–38^. Added as a filler to PCL-based scaffolds, rGO increases the strength, stiffness, and toughness of the scaffolds and produces structures that are more mechanically competent and suitable for load-bearing applications^32,33^. The physicochemical properties of rGO increase the hydrophilicity of various scaffolds and their capacity to adsorb proteins and growth factors^33,34^. Moreover, the addition of rGO improves the substrates for the adhesion, spreading, and proliferation of various cells including stem cells. Of note, rGO incorporation can support the differentiation of progenitor and stem cells into certain lineages, which is especially important for developing biomaterials for tissue regeneration^32,34,35^.

In this work, we fabricated 3D printed PCL-rGO composite scaffolds and evaluated the effects of rGO in modulating the properties of the composite scaffolds. The scaffolds were prepared via extrusion-based additive manufacturing, which enabled us to fabricate structures with high fidelity and resolution. We incorporated various amounts of rGO within the 3D printed scaffolds and evaluated them in regard to their printability, and their structural, morphological, mechanical and biological properties. We demonstrate that the addition of rGO can physically reinforce the 3D printed scaffolds and improve upon the mechanical properties of PCL, while having no adverse effects on the printing process or biocompatibility of the produced composite structures.

## Results and discussion

### Ink preparation and 3D printing

**Figure 1:**
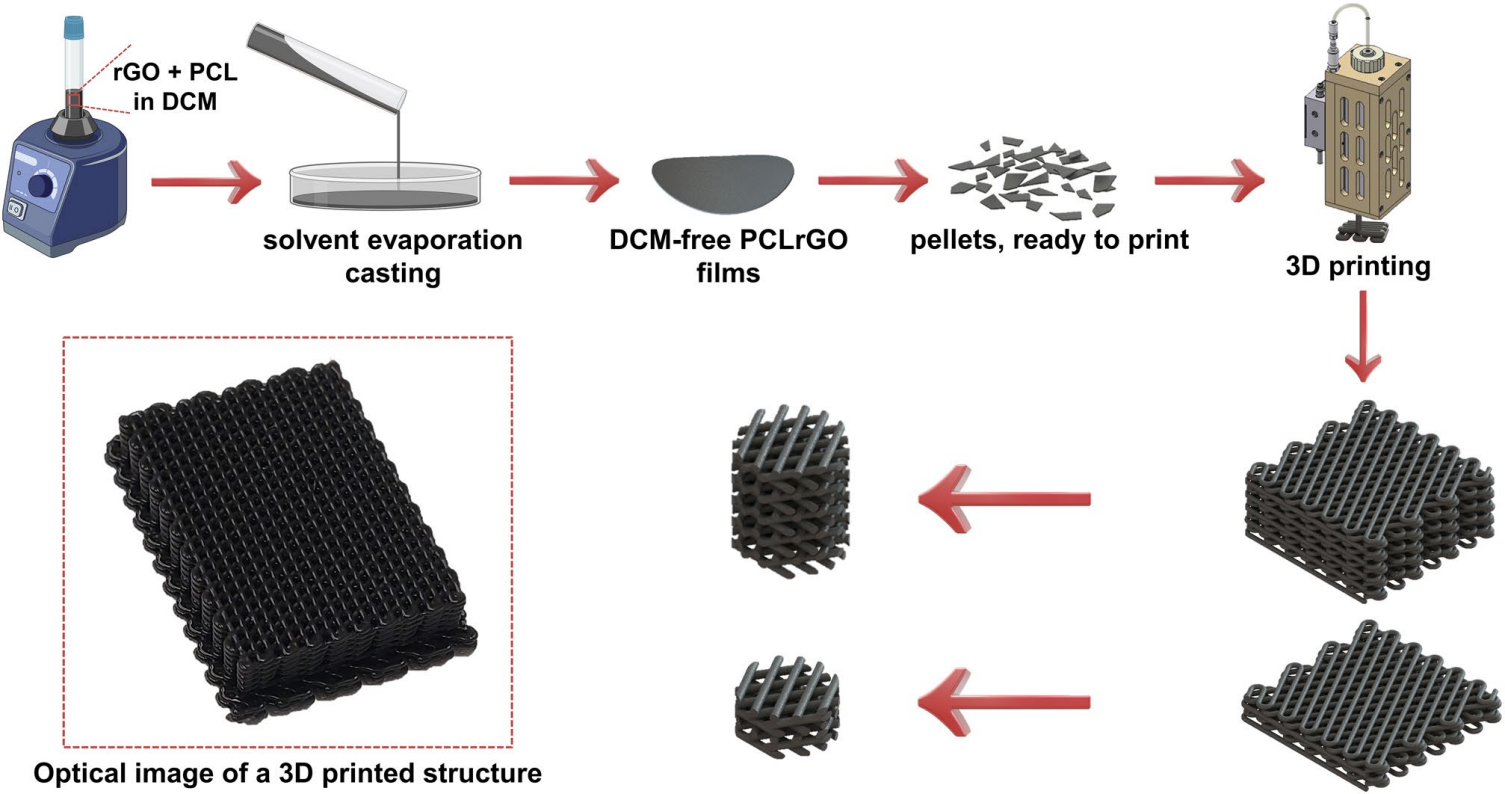
Steps involved in fabricating the composite PCL-rGO 3D printed scaffolds. First, PCL was combined with rGO and vortexed until a homogenous dispersion was achieved. The suspension was then casted into films and the solvent was allowed to evaporate. The collected films were subsequently lyophilized and cut into small pellets to be fed into the 3D printer’s cartridge. Structures of two different thicknesses were 3D printed at a high-temperature and subsequently punched into scaffolds for further assessments. An optical image of the final 3D printed PCL-rGO structure is presented.

Figure 1 shows a schematic overview of the various steps involved in preparing the scaffolds. We employed a two-step fabrication process to ensure an even mixture and distribution of the rGO filler within the 3D printing inks and resultant scaffolds. The 3D printing inks were prepared via solvent evaporation film casting. This technique allowed for the creation of homogenous composites at room temperature circumventing the need to increase the temperature prior to 3D printing. Composite PCL-rGO films of various rGO concentrations (0, 0.5, 1, and 3 wt.%) were prepared beforehand and subsequently cut into smaller pellets that were fed into the high-temperature cartridge of the 3D printer.

To achieve an accurate and consistent pattern in between the scaffolds, the prepared inks were first 3D printed into larger structures and subsequently punched into smaller cylindrical scaffolds. A 60° shift in between subsequent layers was chosen resulting in an interconnected pore structure. The 60° pattern (0/60/120 lay-down pattern) has been previously shown to well support the adhesion^39^, growth, and viability of cells^40^, and have superior mechanical properties^41^ that are anisotropic and independent of the direction of loading^42^.

### Morphological characterization of the 3D printed scaffolds

**Figure 2:**
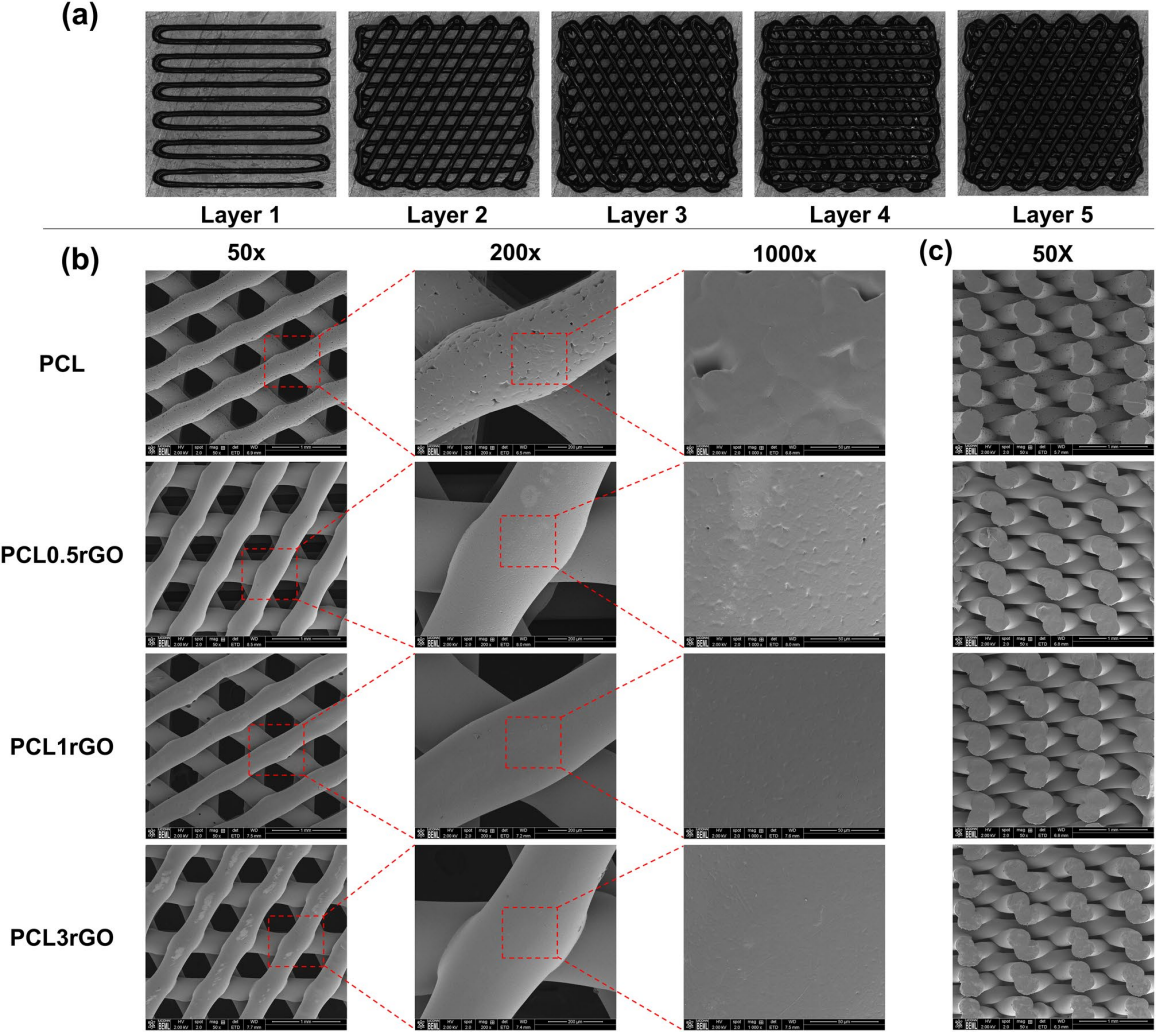
Morphological evaluation of the scaffolds. (**a**) Images taken using the 3D printer’s CCD-camera of each deposited layer during the printing process. (**b**,**c**) SEM images of the 3D printed scaffolds taken from a (**b**) top-view at different magnifications and (**c**) cross-sectional view.

Figure 2 shows the SEM and photo images of the 3D printed scaffolds with various concentrations of rGO. Figure 2a and supplementary video S1 demonstrate the layer-by-layer deposition of the composite inks and the fabrication of the 3D printed structures that were punched into cylindrical scaffolds. The top-view and side-view images in Fig. 2b,c demonstrate the uniformity of the strands and the consistency of the printing process across the different scaffolds. The 50 × top-view images show the 60° shift pattern and the resultant interconnected pores, that are of the same shape and size. The strands are composed of a smooth, dense, and continuous polymer matrix that encompasses rGO. There is a smooth and seamless merging of adjacent layers at the junction between subsequent strands (200 × top-view and 50 × side-view images), ensuring the capacity of the scaffolds to withstand and transfer loads effectively. Moreover, no agglomeration or clumping of the polymer or rGO is detected in any of the high-magnification SEM images, signifying the efficacy of the solvent evaporation casting method in homogeneously dispersing the rGO sheets in the PCL matrix.

**Figure 3:**
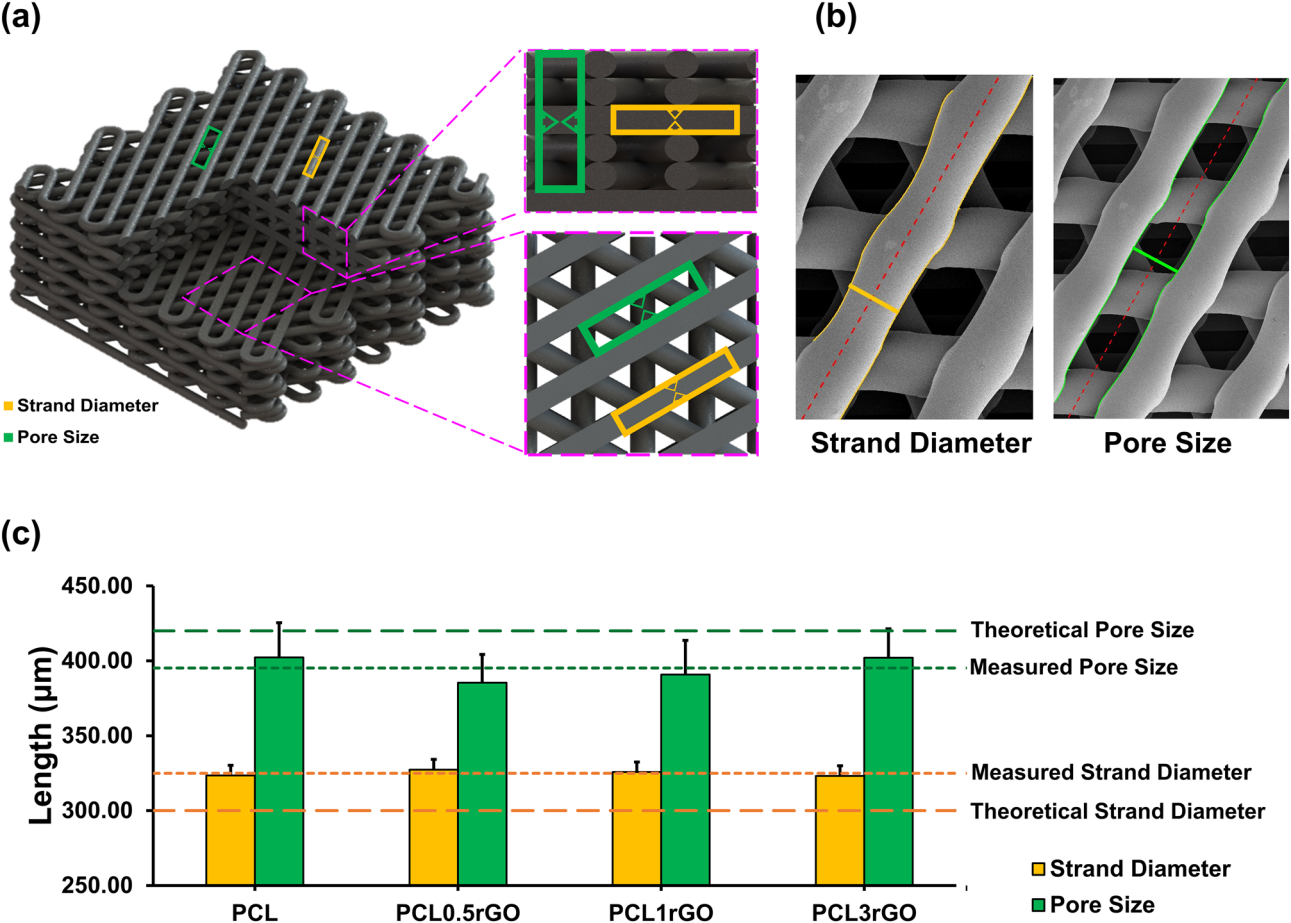
Dimensional characterization of the 3D printed PCL-rGO scaffolds. (**a**) A schematic of the 3D printed structures describing the structure pattern, strand diameter, and pore size from two fields of view. (**b**) A representative image of the measurement process in using the ImageJ macro code. The yellow lines represent the borders of the strands and the green lines represent the borders of the pore areas. The dashed red line indicates the average angle of each strand or pore area. The yellow or green perpendicular line is a representative line of the width that is measured specifying the strand diameter or pore size, respectively. (**c**) The measured strand diameters and pore sizes in comparison to their respective theoretical values. Results are presented as mean ± SD.

The internal architecture of scaffolds is important for guiding cellular functions and new tissue growth and formation. Furthermore, the porosity of a given scaffold governs the seeding, penetration, distribution, and growth of cells^43^. We measured the average strand diameters and pore sizes across all scaffolds (Fig. 3). Theoretical values denote those that were defined prior to printing in the CAD software, SolidWorks. The measured values were obtained through running an ImageJ macro code on the SEM images of the scaffolds post-printing. As shown in Fig. 3c, the average strand diameter and pore size remains similar across all scaffold groups, signifying the unaffectedness of the printed structures by the varying levels of rGO. The ability to manipulate the architecture of composite 3D printed scaffolds through solely manipulating the printing parameters is instrumental in fabricating structures with high fidelity, repeatability, and consistency.

There was a slight variation between the theoretical values and measured values for both the strand diameter and pore size. The measured average strand diameter across all groups was 324.95 ± 1.95 μm, approximately 24.95 μm larger than the theoretical value. This increase in strand diameter is due to the inks die-swelling and expanding upon extrusion from the nozzles, a behavior which is typical of viscoelastic polymer inks^44,45^. The measured average pore size (395.17 ± 8.41 μm) was approximately 24.83 μm smaller than its respective theoretical value. The decrease in pore size is secondary to and almost equal to the expansion in strand diameter, signifying the accuracy of printing (resolution < 1 μm). Importantly, an interconnected pore network with pore sizes in the range of ~ 400 μm is shown to be beneficial for tissue ingrowth and vascularization, especially for bone and cartilage tissue^46,47^.

### Material characterization of the 3D printed scaffolds

**Figure 4:**
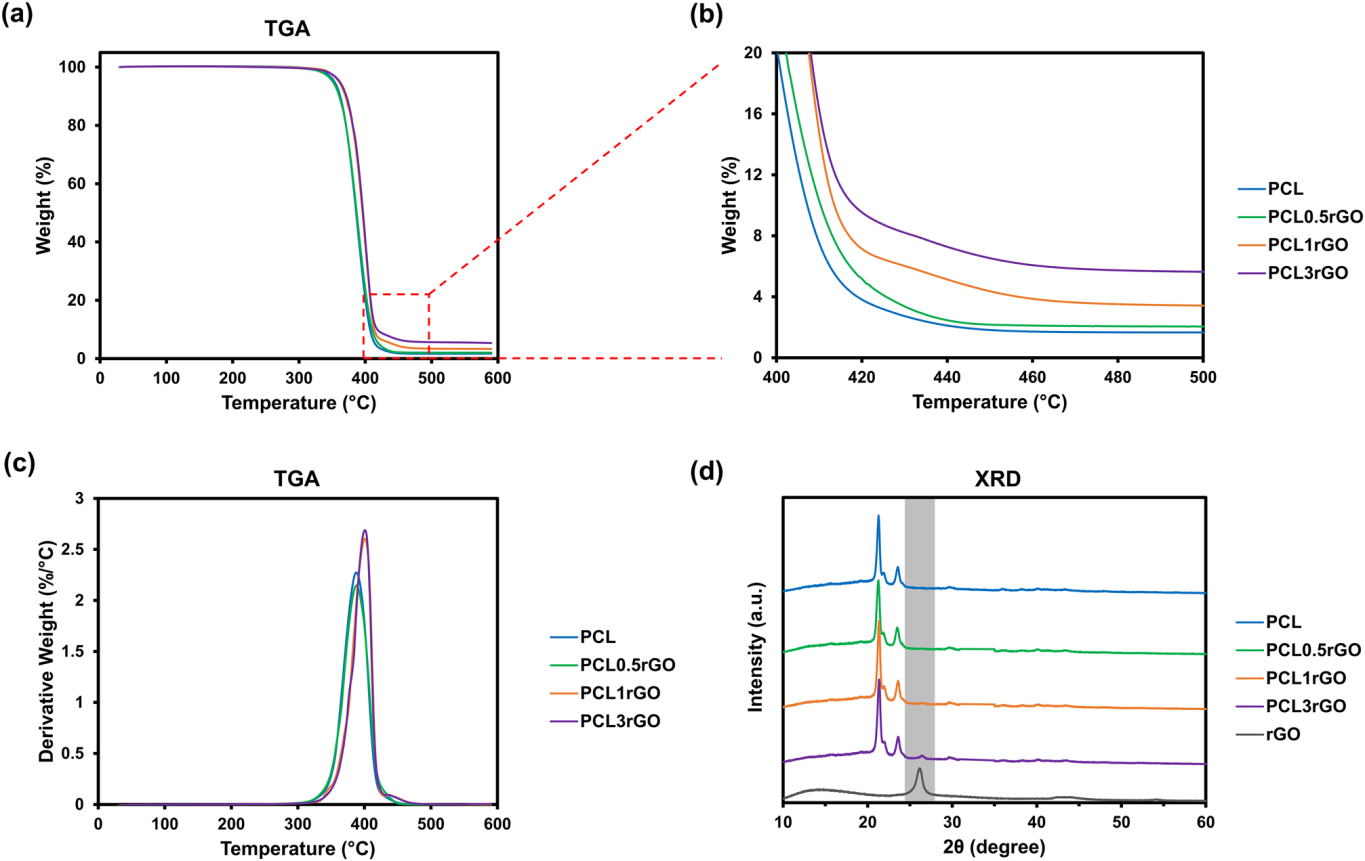
Compositional characterization of the 3D printed scaffolds. (**a**) The TGA thermograms of the 3D printed scaffolds. (**b**) The enlarged region of the TGA thermograms indicating the remaining mass in each sample. (**c**) The TGA first derivative curves of the 3D printed scaffolds. (**d**) The XRD patterns of non-porous 3D printed scaffolds and pure rGO.

Thermogravimetric analysis was used to assess the composition the 3D printed scaffolds and the amount of rGO incorporated within them. This was feasible since PCL and rGO thermally decompose at different temperatures. The TGA curves of all scaffolds are shown in Fig. 4a–c. The sharp drop in mass observed in PCL between 300 to 450 °C corresponds to the structural decomposition of the polymer. This pronounced mass loss was observed in all the composite PCL-rGO samples and corresponds to PCL being the major constituent of the composites. After 450 °C, the remaining mass in all samples was relatively constant and was in direct proportion to the amount of rGO within the samples (Fig. 4b).

X-ray powder diffraction analysis was used to further evaluate the composition of the scaffolds (Fig. 4d). PCL has two characteristic peaks at 2θ = 21.9° and 2θ = 23.5°, which correspond to the (1 1 0) and (2 0 0) planes, respectively^48^. These two peaks were observed in all the 3D printed scaffolds demonstrating the presence of this semi-crystalline polymer in all samples. The characteristic peak of rGO is at 2θ = 26.16°, which corresponds to the (0 0 2) plane^49^. Although rGO was incorporated at smaller amounts within the composites and had a much lower peak intensity than PCL, the distinct peak of rGO was still detectable in the composite structures, especially in PCL3rGO.

**Figure 5:**
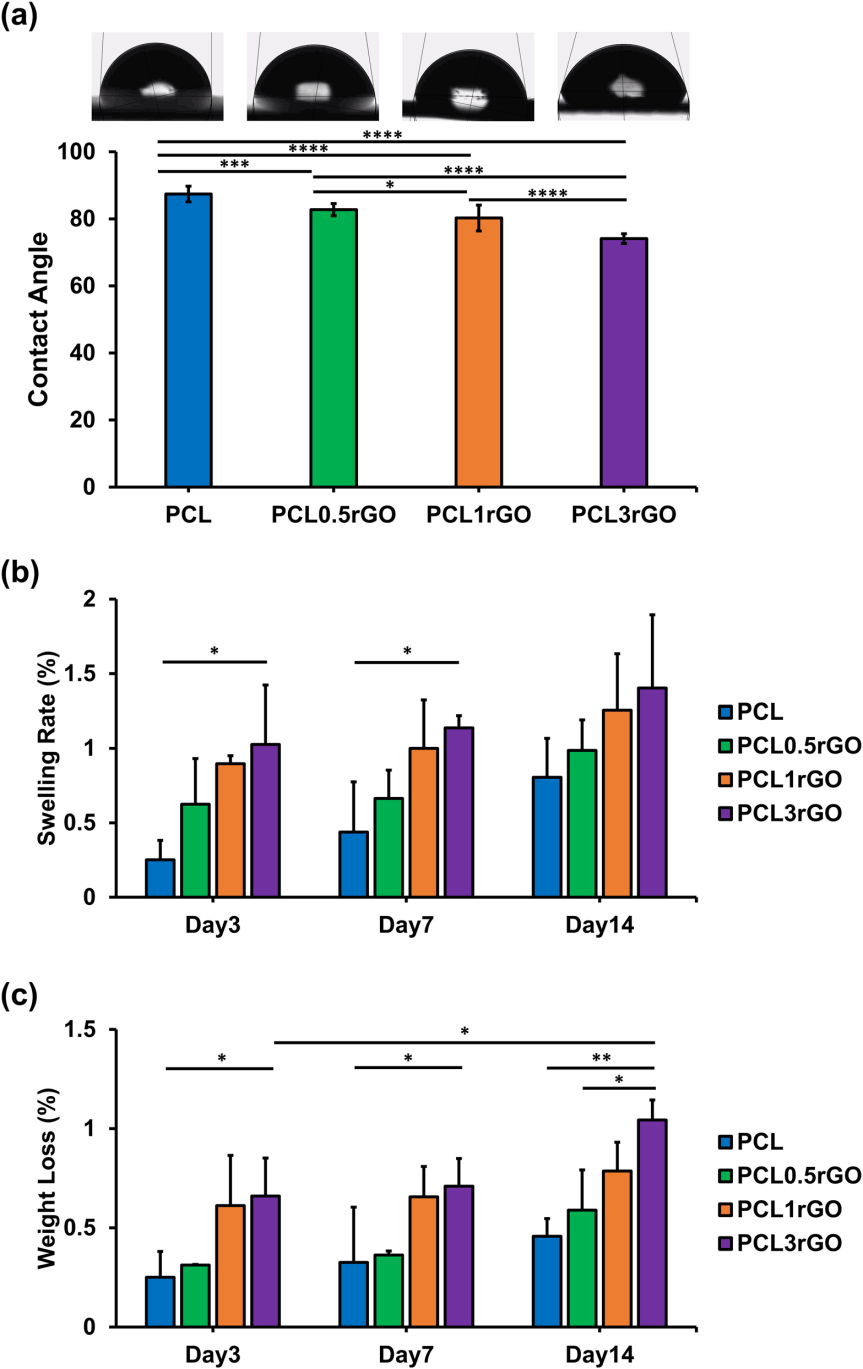
Characterization of the hydrophilicity and in vitro biodegradation behavior of the scaffolds. (**a**) The contact angle images and measurements of the scaffolds with different concentrations of rGO. Results are presented as mean ± SD (n = 5). (**b**,**c**) The in vitro degradation behavior of the 3D printed scaffolds after incubation in SBF over the course of 14 days and under physiological conditions. The (**b**) swelling rate and (**c**) weight loss of the scaffolds expressed in % and presented as mean ± SD (n = 3) (*p < 0.05, **p < 0.01, ***p < 0.001, ****p < 0.0001).

Static contact angle measurements were performed to understand the wettability and hydrophilic/hydrophobic properties of the samples (Fig. 5a and Supplementary video S2). The water contact angle of PCL was measured at 87.4°, indicating its hydrophobic nature. The addition of rGO reduced the water contact angle. There was a continuous decrease in the water contact angle concomitant with the increase in rGO measuring at 82.7°, 80.2° and 74.1° for PCL0.5rGO, PCL1rGO, and PCL3rGO, respectively with statistical significance observed in between all study groups. The capability of rGO and the GFMs, in general, to increase substrate hydrophilicity is well-documented and is considered a critical factor that affects the biocompatibility of graphene-based materials and their interactions with cells^38,50–54^.

To further evaluate the capacity of the PCL-rGO scaffolds for utilization in regenerative engineering applications, their biodegradation behavior was assessed. Scaffolds were incubated in simulated body fluid (SBF) and under physiological conditions for a period of 14 days, after which their swelling rate percentage and weight loss percentage values were measured (Fig. 5b,c). Similar to the trends observed in contact angle measurements, the addition of rGO at all concentrations increased the swelling rate percentage of the PCL-rGO scaffolds compared to bare PCL scaffolds. The increase in swelling rate percentage was in direct proportion to the rGO content of the scaffolds with PCL3rGO scaffolds displaying the greatest water uptake as a function of time (Fig. 5b). The weight loss percentage of the scaffolds similarly corresponded to the results of the contact angle measurements and the swelling rate behavior of the scaffolds. There was an increase in the weight loss percentages of the PCL-rGO scaffolds that was in direct proportion to the rGO content of the scaffolds and the incubation time (Fig. 5c). Thus, the addition of rGO to the PCL-rGO composite scaffolds improved the hydrophilicity of the scaffolds, increased their water uptake and swelling, and accelerated their rate of degradation.

### Mechanical evaluation of the 3D printed scaffolds

**Figure 6:**
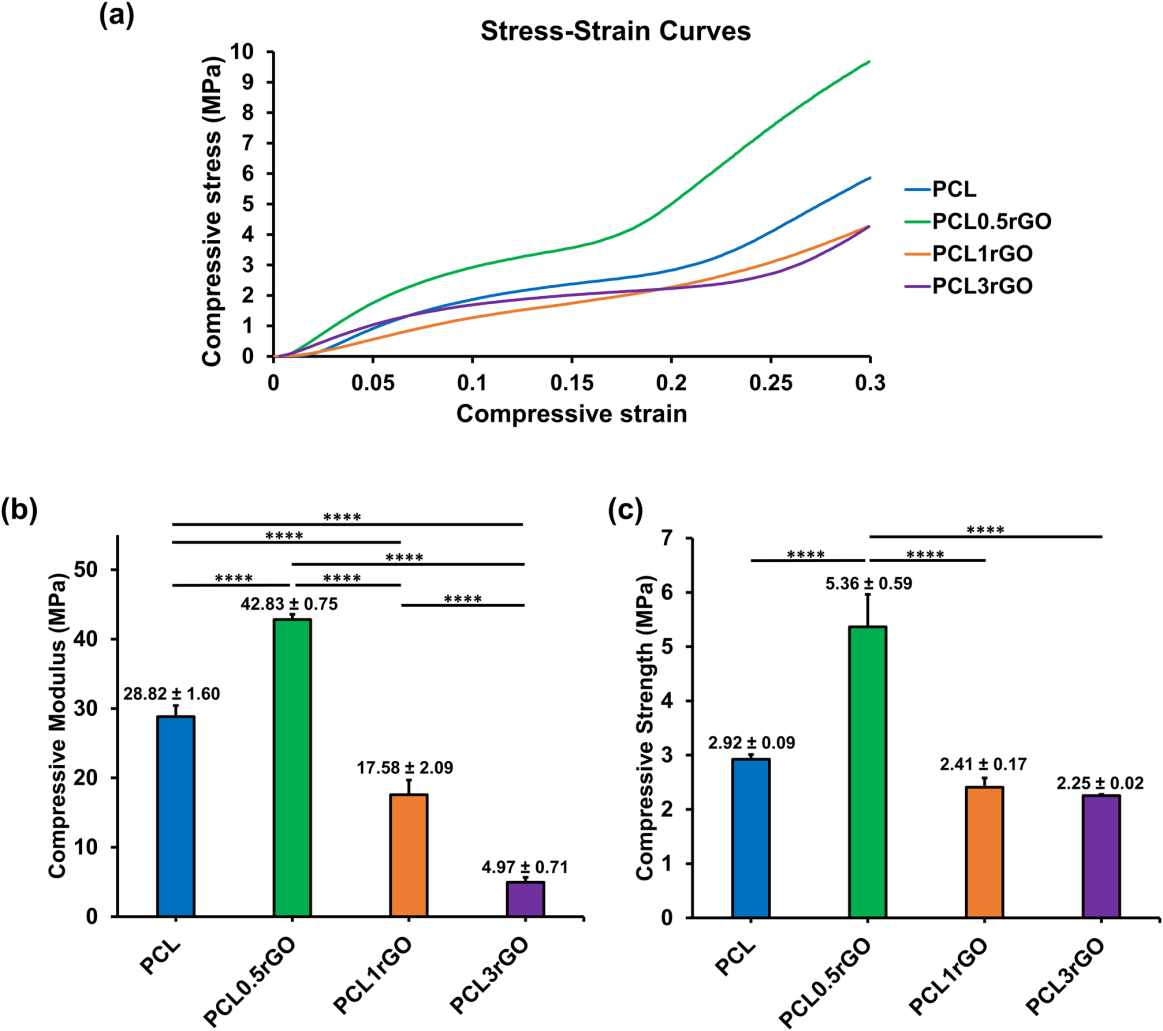
Mechanical analysis of the 3D printed scaffolds. (**a**) The representative stress–strain curves of the 3D printed scaffolds under uniaxial compression loading. (**b**) The compressive moduli and (**c**) compressive strength of the 3D printed scaffolds. Results are presented as mean ± SD (n = 4) (****p < 0.0001).

To evaluate the mechanical performance of the scaffolds and the effects of rGO incorporation, scaffolds of 4 mm thickness × 4 mm diameter were prepared and subjected to uniaxial compressive testing. Figure 6 shows the stress–strain curves of the various scaffolds, and the calculated compressive moduli and strength values. All scaffolds displayed similar stress–strain curves under loading. There is an initial linear elastic or Hookean region followed by a plateau that is further followed by a densification region. As such, the addition of rGO from 0.5–3 wt.% did not affect the deformation behavior of the scaffolds.

As shown in Fig. 6b,c, the addition of rGO at 0.5 wt.% led to the greatest improvements in mechanical properties. There was a significant increase in both the compressive modulus and compressive strength of the PCL0.5rGO scaffolds. Compared to the PCL scaffolds, the compressive modulus and strength of the PCL0.5rGO scaffolds was enhanced by 150% and 185%, respectively. Increasing the rGO content beyond 0.5 wt.%, to 1 wt.% or 3 wt.% reduced the mechanical properties of the scaffolds and deteriorated their mechanical performance.

**Figure 7:**
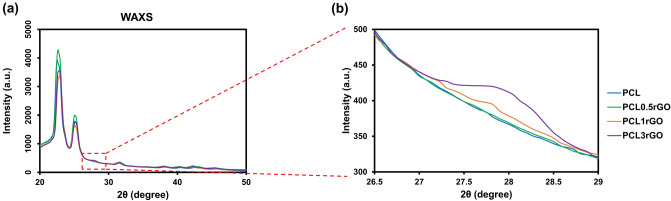
Sub-nanometer level structural analysis of the 3D printed PCL-rGO scaffolds. (**a**) The WAXS patterns of the 3D printed scaffolds. (**b**) The enlarged region of the WAXS pattern indicating the characteristic peak of graphite which corresponds to the aggregation and re-stacking of the graphenic layers.

The mechanisms behind the improved mechanical properties of the scaffolds at 0.5 wt.% rGO was investigated at the sub-nanometer level structure using wide-angle X-ray scattering (WAXS) (Fig. 7). PCL displays two significant peaks at 2θ = 22.5° and 2θ = 25°, which are attributed to its crystalline structure. The characteristic (0 0 2) peak of graphite or aggregated graphenic layers that corresponds to the interlayer distance (d-spacing) of 3.35 Å in between the sheets is at approximately 2θ = 27°^55,56^. The absence of this peak in PCL0.5rGO and its presence in PCL1rGO and PCL3rGO denotes the aggregation or re-stacking of the rGO sheets that is occurring in these samples and only at the higher concentrations of rGO loading whereas at lower concentrations of 0.5 wt.%, the rGO sheets are exfoliated and homogeneously dispersed within the polymeric matrix. The intrinsic high strength of the rGO sheets, their high specific surface area and their homogenous dispersion within the polymer matrix enable strong interfacial interactions to form between the rGO sheets and the polymer chains, which facilitate efficient stress transfer to take place between the two and results in the reinforcing behavior that is observed in the PCL0.5rGO composites. Increasing the rGO filler content beyond 0.5 wt.% in PCL1rGO and PCL3rGO causes the rGO sheets to re-stack and form irreversible aggregates, which impede the efficient transfer of load and diminish the mechanical performance of the scaffolds^57,58^.

The presence of the high-modulus GFMs in low-modulus polymer matrices can lead to significant reinforcements in the mechanical properties of composite structures. The reinforcing effects of the GFMs in polymer matrices is governed by the structure of the graphenic material in use, the polymer matrix, the composite preparation method, and the dispersion of the graphenic filler within the matrix and their interactions^27,59^. In 3D printed scaffolds specifically, in addition to the structural and physicochemical properties of the composite materials, geometrical features such as pore size, pore area, and strand diameter play important roles^50,60^. While most studies have reported that incorporating the GFMs into scaffolds and structures improves their mechanical performance^51,61–65^, a few studies have shown differing results in which the addition of rGO has had no effect or adverse effects on the mechanical performance of the constructs^35,66^. The variations in between studies and the mechanical enhancements observed in our 3D printed constructs, is likely due to variations in all the above-mentioned parameters as well as the differing geometrical features. Importantly, the consistency in strand diameter and pore size across the different PCL-rGO scaffolds (Fig. 3), irrespective of the amount of rGO within the scaffolds, signify that the enhanced mechanical performance of the PCL0.5rGO scaffolds is due to the presence of rGO and not because of any geometrical features. In addition to the two-step fabrication process that was employed to fabricate seamless scaffolds, the larger strand diameters and smaller pore sizes of our constructs also contribute to the improved mechanical behavior. Finally, the homogeneous dispersion of the exfoliated rGO sheets within the polymer matrix is pivotal in the efficient transfer of loads and the reinforcing effects that are optimally observed in PCL0.5rGO.

Lastly, to investigate whether processing PCL through film casting affected its mechanical properties, the elastic moduli of 3D printed scaffolds that were prepared using film-casted PCL pellets was compared to those that were prepared using raw as-is PCL pellets. There was no significant difference between the scaffolds that were printed using either method (Supplementary Fig. S1), suggesting that the procedural steps involved in ink preparation did not influence the mechanical properties of the PCL printed structures.

### In vitro evaluation of the 3D printed scaffolds

The biocompatibility of the scaffolds and their capacity to support cell adhesion and growth is pivotal for tissue and regenerative engineering applications. Thus, the cytocompatibility of the 3D printed PCL-rGO scaffolds and the influence of rGO incorporation was examined using human adipose-derived stem cells (hADSCs). Cells were seeded onto the scaffolds and at pre-determined time points evaluated using the live/dead assay stain and the MTS cellular proliferation assay.

**Figure 8:**
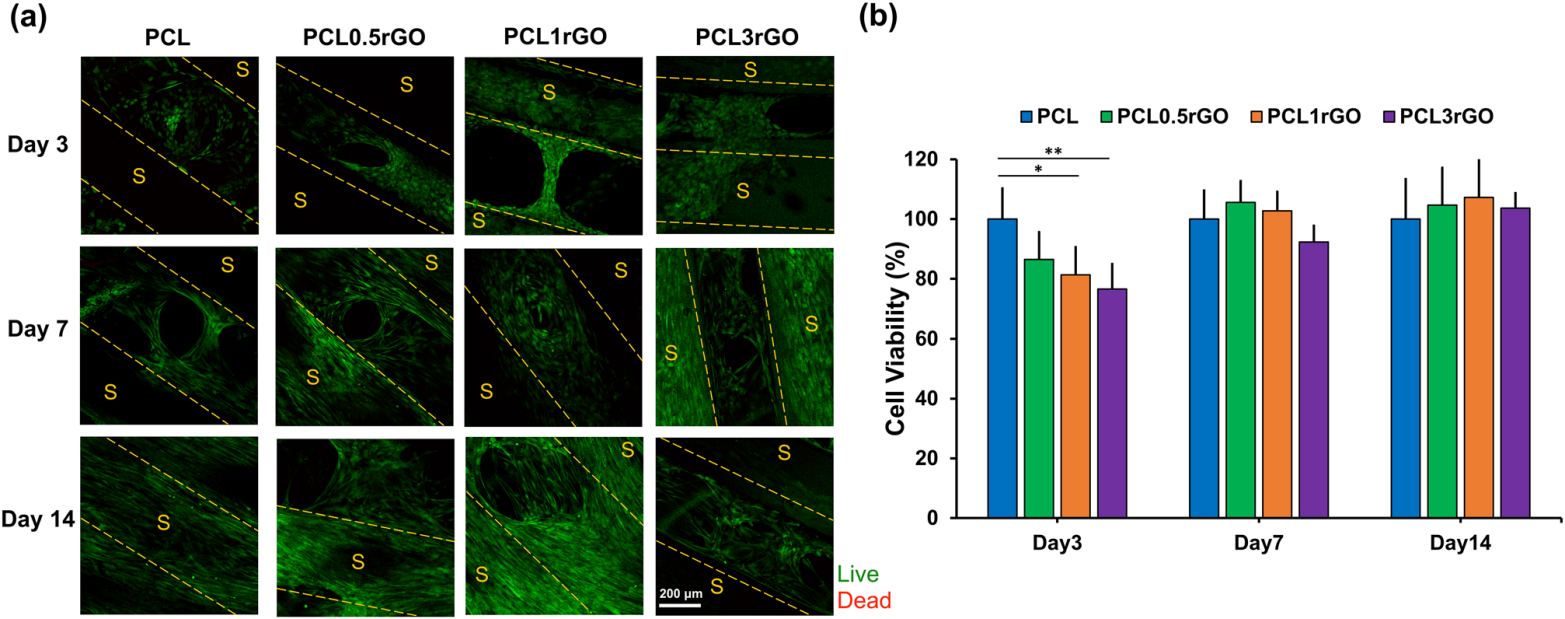
Cytocompatibility of the 3D printed scaffolds. (**a**) Representative confocal micrographs of hADSCs grown on the 3D printed scaffolds and stained with the fluorescent live/dead assay (green, calcein AM; red, ethidium homodimer-1). The strand borders are identified using dashed lines (scale bar 200 μm). (**b**) Cell viability of hADSCs on the 3D printed scaffolds, measured by the MTS assay. Results are expressed as % viability with respect to the PCL control at each time point and are presented as mean ± SD (n = 3) (*p < 0.05, **p < 0.01).

Figure 8a shows the representative confocal images of the cell-seeded scaffolds at days 3, 7, and 14. There was high cell viability in scaffolds of all compositions with minimal to no cell death. All scaffolds promoted the adhesion and growth of the hADSCs, irrespective of rGO content. The addition of rGO appeared to promote the growth and proliferation of cells to a greater extent than the PCL scaffolds. The PCL0.5rGO and PCL1rGO scaffolds, in specific, had a greater coverage of cells both on the 3D printed strands and pore regions in between (Fig. 8a and Supplementary Fig. S2). Additionally, there was greater cellular bridging in between the strands, signifying the superior biological activity of the cells on these scaffolds.

The presence and proliferation of the hADSCs on the 3D printed scaffolds was further quantified using the MTS proliferation assay, and through using PCL scaffolds as controls (Fig. 8b). There was a significantly lower number of cells on the composite scaffolds at day 3 post-seeding in a concentration-dependent manner. This is likely due to the greater hydrophilicity of the PCL-rGO scaffolds compared to the PCL scaffolds, which upon initial cell seeding permits the cell droplets to penetrate and pass through the scaffolds and onto the well plates more easily as opposed to the more hydrophobic PCL scaffolds which retain the droplets on their surface, allowing sufficient time for the cells to attach onto the substrates. At day 7, however, the cells on the PCL-rGO composite scaffolds had proliferated and compensated for the initial cell deficiency. There was a slightly higher trend in the percentage of viable cells at days 7 and 14, however no significant difference was detected at either time point between any of the study groups.

The biocompatibility of the GFMs is regulated by their physicochemical characteristics (size, shape, surface chemistry), exposure conditions (medium, dose, duration), and the cell type^25,27^. Most studies have shown that the incorporation of small amounts of the GFMs into scaffolds helps to improve their biological performance^67–69^. The addition of the GFMs improves surface roughness, wettability, and protein adsorption leading to increased cell adhesion, proliferation, and differentiation into certain lineages. The non-covalent π–π stacking and the presence of the oxygen functional groups in rGO along with their high specific surface area allow for the high density loading and adsorption of proteins which in turn facilitate cell adhesion, spreading, and proliferation^70^. The cellular adhesion and sustained viability and growth of the hADSCs on the composite PCL-rGO scaffolds provides evidence for the biocompatibility of these substrates. The addition of rGO at all of the tested concentrations was well-tolerated by the cells and had no adverse effects.

## Conclusion

In summary, we have prepared 3D printed PCL-rGO scaffolds that are mechanically enhanced and biologically compatible. We employed a two-step fabrication process to ensure the homogeneous mixture and distribution of rGO within the PCL matrix and to obviate the need for increasing the temperature prior to printing. The resultant scaffolds were 3D printed with high fidelity, repeatability, and consistency. The homogenous distribution of the rGO sheets within the PCL matrix and the design of the constructs led to significant improvements in the mechanical properties of the resultant scaffolds. The addition of rGO, at small amounts of 0.5 wt.%, significantly improved the compressive modulus and compressive strength of the scaffolds leading to scaffolds that were mechanically more competent. Conversely, increasing the rGO filler content beyond 0.5 wt.% diminished the mechanical enhancements and led to the deterioration of the mechanical properties, due to the re-stacking and aggregation of the rGO sheets within the matrix. Of importance, the addition of rGO had no adverse cellular effects and all scaffolds were shown to well support the growth and viability of stem cells in vitro. In sum, through combining small amounts of rGO as a filler within PCL, as one of the most widely used polymers in scaffold fabrication, we have prepared 3D printed composite scaffolds that are both mechanically reinforced and biologically compatible. The PCL-rGO scaffolds presented in this study afford unique opportunities for the regenerative engineering of various tissues and organs.

## Materials and methods

### Ink preparation

The 3D printing inks were prepared using a solvent evaporation film casting method. Polycaprolactone (PCL, Mw 43–50 kDa, Polysciences, Warrington, PA), either alone or mixed with reduced graphene oxide (rGO, Sigma-Aldrich) at 0.5, 1 or 3 wt. % was dissolved in dichloromethane (DCM, Sigma Aldrich, St. Louis, MO). The weight of PCL or PCL + rGO to DCM was 1 g to 5 mL. The mixtures were vortexed at room temperature for 3 h to ensure the complete dissolution of PCL and the homogenous dispersion of rGO. The PCL or composite PCL-rGO films were prepared by casting the suspensions onto 100 mm glass petri dishes that were left to air-dry overnight. The films were subsequently collected and lyophilized for another 24 h. Lastly, the films were cut into smaller pellets for feeding into the cartridge of the 3D printer (Fig. 1).

### Scaffold 3D printing

Structures of different thicknesses were designed using a computer-aided design (CAD) software (SolidWorks Version 2018, Dassault Systémes, Vélizy-Villacoublay, France) and exported as STL files into the Perfactory RP software Version 3.0 (EnvisionTEC GmbH, Germany). The designs were sliced into 300 μm thick layers and the angle between subsequent layers was set to 60°. The strand diameter and pore size (defined as the distance between two adjacent strands) were set as 300 μm and 420 μm, respectively (Fig. 3a).

Structures were 3D printed using an extrusion-based 3D printer (4th Generation 3D Bioplotter, Manufacturer Series, EnvisionTEC GmbH, Germany). For this, the stainless-steel high-temperature cartridge was filled with pellets, of either pure PCL or composite PCL-rGO. The temperature was subsequently raised to 100 °C and maintained for the duration of printing. The cartridge was kept at 100 °C for at least 20 min before printing to ensure the complete melting of the pellets and avoidance of air bubbles. The temperature of the platform was kept at 10 °C during the entire process. A pressure of 0.6 MPa and a head movement speed of 1.4 mm/s was used to extrude the inks from of the G24 metallic nozzle. There was a 20 s delay set between the printing of consecutive layers to allow time for sufficient solidification of the previous layers. Once printed, the structures were readily collected. The structures were then punched using disposable punches to create cylindrical scaffolds for further use. Mechanical testing and SEM imaging were performed using scaffolds of 4 mm thickness × 4 mm diameter. All other experiments were performed using scaffolds of 1 mm thickness × 3.5 mm diameter (Fig. 1). All samples were stored in vacuum desiccators post-printing.

### Morphological characterization

In addition to the images taken of each layer of the structure during the printing process with the built-in high definition CCD-camera of the Bioplotter, SEM was used to evaluate the morphological features of the printed scaffolds. The samples (*n* = 3/ group) were mounted onto stainless-steel stubs, sputter coated with gold–palladium, and imaged using a FEI Nova NanoSEM 450 at a working distance of 5 mm and an acceleration voltage of 15 kV.

The average strand diameter and average pore size for each scaffold was measured using a macro code within ImageJ^71^ Version 1.53c (https://imagej.nih.gov/ij/). With this code, first a region of interest consisting of any given strand was selected. The borders of that strand were identified using the code. Perpendicular lines from one border toward the other were measured by the software. The average of these measurements for any strand represents the average width of that strand (strand diameter). This process was repeated until the average width of all strands across all SEM images was measured (average strand diameter) (Fig. 3c). The pore size values were similarly determined by selecting the width between every two adjacent strands as the region of interest.

### Thermogravimetric analysis (TGA)

TGA (TGA Q500, TA Instruments) was used to evaluate the incorporation of rGO within the scaffolds. TGA was performed under nitrogen over a temperature range from room temperature to 600 °C with a ramp rate of 10 °C min^−1^.

### X-ray powder diffraction (XRD)

XRD (Bruker D2 Phaser) was used to identify the presence of rGO within the scaffolds. Samples were loaded into the corundum plates and X-ray diffraction was measured over a range of 2θ = 10–60° with a step size of 0.01°.

### Wettability assessment

The water-in-air contact angle of the samples was measured using a Dataphysics OCA20 contact angle analyzer and the sessile drop method at room temperature. Samples with a flat structure were 3D printed and a drop of 3 µL of deionized water was automatically placed onto them (*n* = 5). The absorption of water was recorded using a high-speed framing camera, and the measurements were performed using ImageJ software Version 1.53c (https://imagej.nih.gov/ij/).

### Degradation behavior

The degradation behavior of the scaffolds was evaluated by measuring the percentages of weight loss and swelling rate of the study groups over a period of 14 days, when immersed in SBF. The SBF solution was prepared following previously described methods^72^. Briefly, 7.996 g of NaCl, 0.350 g of NaHCO_3_, 0.224 g of KCl, 0.228 g of K_2_HPO_4_.3H_2_O, 0.305 g of MgCl_2_.6H_2_O, 40 mL of 1 M-HCl, 0.278 g of CaCl_2_, 0.071 g of Na_2_SO_4_, and 6.057 g of (CH_2_OH)_3_CNH_2_ (all purchased from Sigma) were sequentially dissolved in 1 L of distilled water that was maintained at 36.5 °C. After all salts were dissolved and 1–2 min after the addition of the last salt, the pH of the solution was adjusted to ~ 7.4. The initial weights of the scaffolds (*n* = 3/ group/ timepoint) were recorded. The scaffolds were then incubated in a 15 mL solution of 1 × SBF and kept under gentle agitation at 37 °C. The SBF solution was prepared fresh weekly. The scaffolds were harvested at days 3, 7, and 14 for further assessments.

The swelling behavior of the scaffolds was assessed by placing the harvested scaffolds onto a filter paper under vacuum for 1 min and by recording their wet weights. The scaffolds were then freeze-dried for 48 h, and their dry weights were recorded. The swelling rate percentage was determined following the equation:

$$Swelling\, rate= \frac{{W}_{w}-{W}_{d}}{{W}_{d}} \times 100$$

where *W*_*w*_ is the wet weight, and *W*_*d*_ is the dry weight.

The weight loss percentage was determined using the equation:

$$Weight\, loss= \frac{{W}_{i}-{W}_{d}}{{W}_{i}} \times 100$$

where W_i_ is the initial weight, and W_d_ is the dry weight^73^.

### Mechanical studies

The mechanical properties of the scaffolds were evaluated using the Instron 5544 mechanical tester (Norwood, MA) equipped with a 2 kN load cell. Scaffolds (*n* = 4/ group) were subjected to uniaxial compressive loading that was carried out at a crosshead speed of 2 mm/min to a strain limit of 30%. Measurements were calculated using the Bluehill Universal software Version 3.61. The elastic modulus was determined using the 0.2% strain offset linear slope method and the compressive strength values were determined based on compressive stress at 20% strain.

### Wide-angle X-ray scattering (WAXS)

The WAXS patterns were acquired using an Oxford Diffraction XCalibur PX Ultra (Concord, MA) with an Onyx CCD area detector and Cu-Kα radiation. The scaffolds were mounted using a custom-made sample holder and measurements were made over a range of 0 to 50 with a step size of 0.03 2θ at room temperature. Data were collected and analyzed using CrysAlisPro software Version 171.40.67a. The magnitude of the scattering vector was calculated using the equation below:

$$q=\left(\frac{4\pi }{\uplambda }\right)sin\frac{\uptheta }{2}$$

where θ is the scattering angle and λ = 1.5418 Å is the X-ray wavelength for Cu-Kα.

### In vitro studies

Human adipose-derived stem cells (hADSCs) and all cell culture reagents were purchased from Invitrogen (Carlsbad, CA). The CellTiter 96 Aqueous One Solution Cell Proliferation Assay (MTS) was purchased from Promega (Madison, WI). The hADSCs were grown in MesenPRO RS basal medium with 0.5% penicillin/streptomycin, 1% L-glutamine and 2% MesenPRO RS growth supplement with regular media changes every 2 or 3 days. At P2, the cells were detached from the culture flasks using TrypLE Express without phenol red, centrifuged at 210*g* for 5 min, and seeded onto sterile scaffolds that had been placed in ultra-low attachment 96-well plates. The scaffolds had been previously sterilized by immersion in 70% ethanol for 20 min, followed by UV exposure for 30 min. The cells were seeded at a density of 50,000 cells/scaffold in 5 μL of media and allowed to attach to the scaffolds for 1 h before adding growth media to the wells.

#### Live/dead assay

The viability of the cells on the 3D printed scaffolds was evaluated using the LIVE/DEAD assay kit at days 3, 7 and 14. The scaffolds were transferred into new wells, rinsed twice with DPBS, and then incubated in a staining solution (10 mL DPBS, 5 μL calcein AM, and 20 μL ethidium homodimer-1) for 15 min. Imaging was carried out using confocal microscopy (Zeiss LSM Confocor2 at × 10 magnification). Afterwards the ImageJ software Version 1.53c (https://imagej.nih.gov/ij/) was used to apply a 3D Median filter to all the images during the z-projection process to reduce the background noise that was due to the presence and interference of the PCL polymer.

#### Viability

The viability and proliferation of the cells on the 3D printed scaffolds was evaluated using the MTS assay at days 3, 7 and 14. The scaffolds (*n* = 3/group) were transferred into new wells, washed once with DPBS, and then incubated in the MTS solution (30 μL MTS reagent, 200 μL growth medium) for 2 h at 37 °C. The absorbance values of the samples were subsequently read in triplicate at 490 nm using a plate reader (BioTek, Synergy H1, Winooski, VT). To negate the interference of the MTS reagent with the scaffolds, scaffolds with no cells were used and their absorbance values were deducted from that of cell-seeded scaffolds. The data from each time point was normalized to the mean absorbance values of the control PCL scaffolds of that time point, and reported as cell viability (% of control).

### Statistical analysis

All experimental data are presented as mean ± standard deviation (SD). Statistical analysis was performed using one-way analysis of variance (one-way ANOVA) using the Holm-Sidak post hoc test and two-way analysis of variance (two-way ANOVA) using Bonferroni’s post hoc test. Significant levels are determined at *p < 0.05, **p < 0.01, ***p < 0.001 and ****p < 0.0001.

## Supplementary Information

Supplementary Video 1.

Supplementary Video 2.

Supplementary Information 1.
