# Supplementary material for: Fabrication and characterization of mechanically competent 3D printed polycaprolactone-reduced graphene oxide scaffolds
Source: Sci Rep. 2020 Dec 17;10:22210. doi: 10.1038/s41598-020-78977-w (PMC7747749; doi:10.1038/s41598-020-78977-w)
Supplement: Supplementary file 3 — Supplementary Information 1. [file 41598_2020_78977_MOESM3_ESM.docx]

**Fabrication and characterization of mechanically competent 3D printed polycaprolactone-reduced graphene oxide scaffolds**

Amir Seyedsalehi^1,2,3,4,*^, Leila Daneshmandi^1,2,3,4,*^, Mohammed Barajaa^1,2,3,4^, John Riordan^1,2,3^, Cato T. Laurencin^1,2,3,4,5,6,7,#^

^1^Connecticut Convergence Institute for Translation in Regenerative Engineering, UConn Health, Farmington, CT 06030, USA

^2^Raymond and Beverly Sackler Center for Biomedical, Biological, Physical and Engineering Sciences, UConn Health, Farmington, CT 06030, USA

^3^Department of Biomedical Engineering, University of Connecticut, Storrs, CT 06269, USA

^4^Department of Orthopaedic Surgery, UConn Health, Farmington, CT 06030, USA

^5^Institute of Materials Science, University of Connecticut, Storrs, CT 06269

^6^Department of Materials Science and Engineering, University of Connecticut, Storrs, CT 06269, USA

^7^Department of Chemical and Biomolecular Engineering, University of Connecticut, Storrs, CT 06269, USA

* These authors contributed equally to this work

**#Corresponding author:**

*Cato T. Laurencin, MD PhD*

Connecticut Convergence Institute,

UConn Health,

293 Farmington Avenue

Farmington, CT 06030

Email: Laurencin@uchc.edu

Tel.: 860-679-6600

Fax: 860-679-1553

**Supplementary Info**


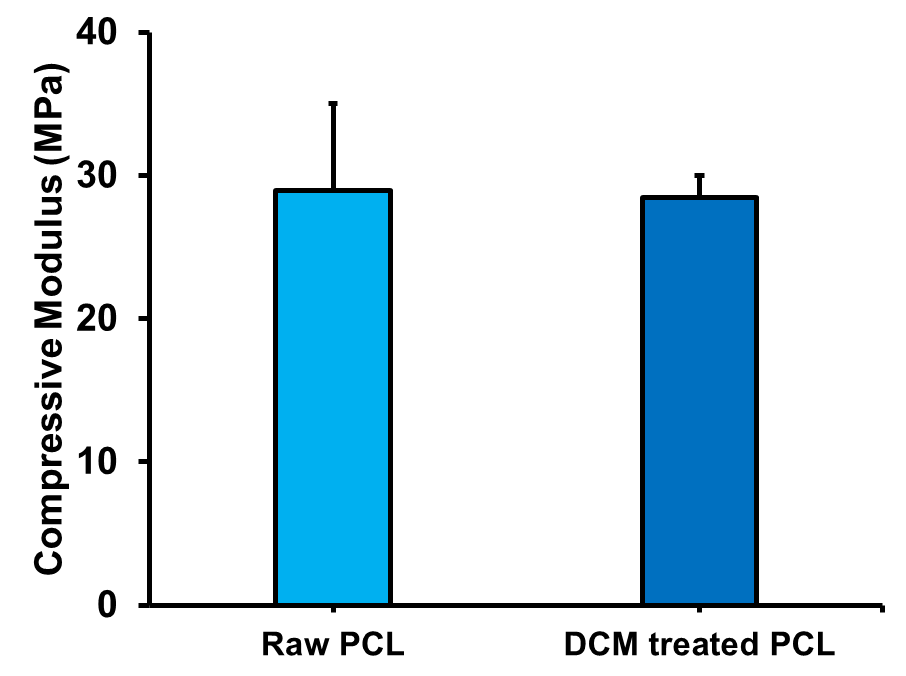


Figure S1. The compressive moduli of 3D printed scaffolds prepared from raw PCL pellets and PCL films. Results are presented as mean ± SD.


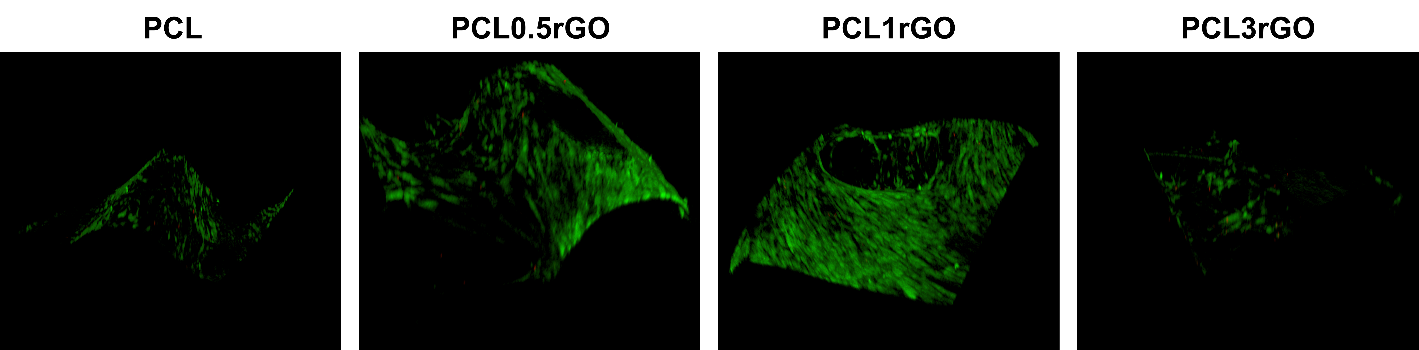


Figure S2. Representative 3D reconstructions of the confocal micrographs showing the presence and distribution of the hADSCs throughout the scaffold strands and pore areas at day 14. Cells were stained with the fluorescent live/dead assay (green, calcein AM; red, ethidium homodimer-1).

Video S1. Different layers of the 4 mm thick 3D printed structure, during the printing process.

Video S2. Contact angle test of representative samples from each group of study.
